# Expanding the Applicability of Cognitive Charts to the Entire Age Span

**DOI:** 10.3390/brainsci15040327

**Published:** 2025-03-21

**Authors:** Christian Gourdeau, Marie-Pierre Légaré-Baribeau, Patrick Bernier, Robert Laforce

**Affiliations:** 1 Département de Physique, Cégep Limoilou, Quebec City, QC G1J 5L5, Canada; christian.gourdeau@cegeplimoilou.ca; 2 Département des Sciences Neurologiques, Clinique Interdisciplinaire de Mémoire (CIME), CHU de Québec, Québec, QC G1J 1Z4, Canada; mp.legare.baribeau@gmail.com; 3 Services Gériatriques Spécialisés, CIUSSS de la Capitale-Nationale, Quebec City, QC G1M 2R9, Canada; patrick.bernier.med@ssss.gouv.qc.ca

**Keywords:** cognitive assessment, screening tools, age-associated cognitive decline, mild cognitive impairment, Alzheimer’s disease

## Abstract

**Background/Objectives:** We previously developed Cognitive Charts (CCs) for early detection and/or longitudinal evaluation of age-associated cognitive decline on widely used cognitive screening measures such as the Mini-Mental State Examination (CC-MMSE) and the Montreal Cognitive Assessment (CC-MoCA). Similar to growth curves used in Pediatrics, clinicians can quickly interpret an individual’s performance on the MMSE or MoCA, track the patient’s longitudinal cognitive trajectory, and subsequently intervene earlier based on the findings (see quoco.org). This has proven very helpful to frontline clinicians, particularly in light of the newly approved monoclonal antibodies for treatment of Alzheimer’s disease. To this date, however, the CC-MMSE and CC-MoCA only applied to limited age ranges. We validated herein our CCs across the entire age span. **Methods:** Two datasets were obtained from the National Alzheimer’s Coordinating Center, for a total of 32,560 individuals. We examined average MMSE and MoCA scores for younger individuals compared to the current age thresholds and ensured consistency of age-related Cognitive Quotient scores. **Results:** In this study, both MMSE and MoCA scores show very little variation below the age threshold. If the age is fixed at the threshold in the QuoCo calculation, the resulting score remains constant within this range. Furthermore, CCs performed similar or better in younger individuals. **Conclusions:** Our findings again emphasize the clinical significance of CCs as a tool for monitoring cognitive changes across the entire age span, hence maximizing early detection and appropriate treatment monitoring.

## 1. Introduction

Cognitive screening is vital in primary care medicine to capture early cognitive changes associated with amnestic mild cognitive impairment (MCI) and mild dementia [1]. In recent years, our Cognitive Charts (CCs) have gained momentum as a useful tool and frontline physicians are increasingly using CCs to position and interpret an individual’s performance based on age, education, and score on the Mini-Mental State Examination (CC-MMSE) [2,3] or the Montreal Cognitive Assessment (CC-MoCA) [4,5]. Similar to growth curves used in Pediatrics, clinicians can quickly interpret an individual’s performance on the MMSE or the MoCA using the CCs, track the patient’s longitudinal cognitive trajectory, and subsequently intervene earlier based on the findings (see quoco.org). The most recent Canadian Consensus Conference on Diagnosis and Treatment of Dementia recommends the use of CCs for longitudinal evaluation and follow-up of cognitive performance over time [6]. This can play a major role since recent data suggest that newly approved monoclonal antibodies for Alzheimer’s disease (AD) are more efficacious if administered early [7,8].

### 1.1. Development of Cognitive Charts for the MMSE and MoCA

Cognitive Charts were developed to improve the early detection and longitudinal monitoring of cognitive decline using the MMSE and the MoCA. By integrating age and education level as key factors, they provide a more precise alternative to traditional fixed cutoffs.

The development of CCs is based on linear regression models applied to large longitudinal databases, allowing for characterization of normal cognitive score trajectories according to age and education. Two fundamental concepts emerged from this modeling: the Cognitive Quotient (QuoCo) and Standardized Age. QuoCo is an age-adjusted score, calculated by dividing the cognitive test result by the patient’s age and multiplying it by 1000 to avoid decimal values. Standardized Age, on the other hand, adjusts chronological age to better reflect expected cognitive performance based on education level. This adjustment is made by subtracting a factor related to the number of years of schooling from the actual age. Data analysis revealed that QuoCo follows a normal decline that is linearly associated with Standardized Age, making it a valuable tool for longitudinal cognitive performance monitoring.

To differentiate normal cognitive aging from pathological decline, CCs were designed as percentile curves, similar to pediatric growth charts, allowing for each individual to be positioned relative to a normative distribution. A threshold zone was defined to identify abnormal performances, and the Youden Index was used to optimize the balance between sensitivity (SE) and specificity (SP). This approach enhances clinical monitoring accuracy and facilitates early detection of cognitive decline trajectories.

In 2017, this methodology was applied to the MMSE using data from 7569 participants in the Canadian Study of Health and Aging, followed for 10 years [9]. The constructed model, predicting MMSE scores based on age and education, led to the development of the CC-MMSE, which was validated on an independent sample of 6501 participants from the National Alzheimer’s Coordinating Center—Uniform Data Set (NACC-UDS).

**Figure 1:**
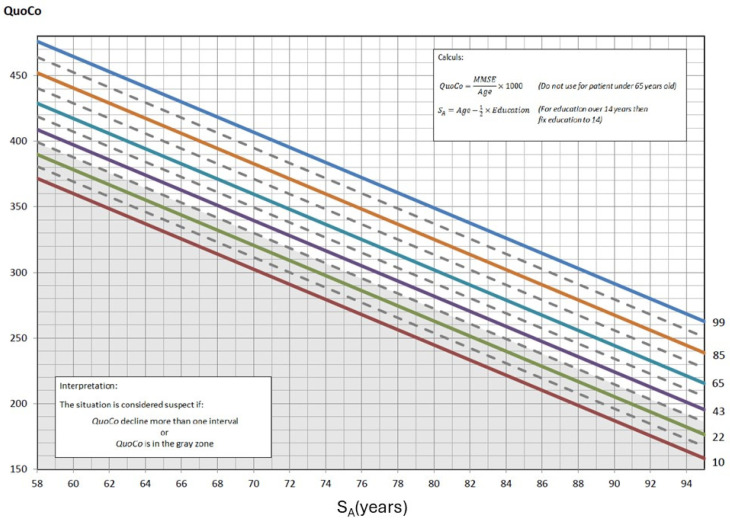
The Cognitive Chart MMSE (CC-MMSE). Each solid parallel line represents a percentile: 99th percentile (blue), 85th percentile (light brown), 65th percentile (turquoise), 43rd percentile (purple), 22nd percentile (green), and 10th percentile (red). These colors were chosen from color palettes for color blindness. The grey zone at the bottom represents the cutoff zone. Points on the chart at baseline that fall within the cutoff zone (or any single point within that zone) or, subsequently, that represent a decline greater than the allotted width of 1 percentile zone from the initial measurements, indicate potential cognitive problems. Note: QuoCo, Cognitive Quotient; SA, Standardized Age [3].

The CC-MMSE was generated by defining curves spaced according to an optimal rate of cognitive decline, maximizing SE and SP through the Youden Index. A threshold zone was integrated to identify at-risk individuals from the first measurement, and longitudinal follow-up showed that a decline exceeding an interval of one percentile line between two successive measurements is an indicator of potential cognitive impairment. In the CC-MMSE, Standardized Age was established by subtracting half the years of education from the actual age. To better visualize what Cognitive Charts represent, refer to Figure 1, which illustrates the cognitive curves developed for interpreting MMSE scores. These percentile-based curves allow clinicians to assess cognitive performance relative to age and education level, enhancing diagnostic accuracy. Clinical case vignettes are illustrated in the Supplementary Material (see Supplementary S1).

In 2022, a similar approach was applied to the MoCA, which has demonstrated greater sensitivity for detecting MCI and superior test–retest reliability compared with the MMSE. The analysis of 9684 participants from the NACC-UDS led to the development of the CC-MoCA, which was validated using several independent databases, including the Alzheimer’s Disease Neuroimaging Initiative and the Hudon database.

**Figure 2:**
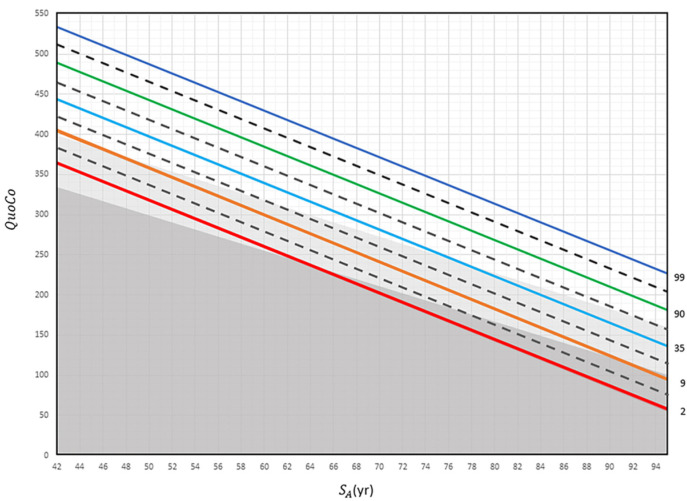
The Cognitive Chart MoCA (CC-MoCA). Standardized Age (SA) is plotted in relation to Cognitive Quotient (QuoCo). Each percentile line (indicated far right) represents expected age-related decline on the Cognitive Chart MoCA scores and is spaced to detect abnormal cognitive changes. A decline of more than any half interval (equivalent to the distance between a full and dashed line) but less than any full interval could be used to detect changes in one step of cognition (normal to MCI or MCI to dementia) and a decline of more than any one full interval (equivalent to the distance between two full lines) to detect dementia. Percentile lines are colored for easy read only. Gray zones discriminate status: normal (white), MCI (light gray), and dementia (dark gray). These charts cannot be used for patients younger than 60 years [3].

This model classifies individuals into different categories: Age-Associated Cognitive Decline (AACD), MCI, and dementia. A decline greater than a half-percentile interval signals a change in cognitive status (e.g., Healthy controls → MCI), while a full-interval decline indicates a transition to dementia. In the CC-MoCA, Standardized Age is determined by subtracting the years of education from the actual age. See Figure 2, which illustrates the CCs developed for interpreting MoCA scores. Clinical case vignettes are presented in the Supplementary Material (see Supplementary S2).

### 1.2. Advantages of Cognitive Charts

CCs offer several major advantages over traditional methods. They allow for precise longitudinal monitoring of cognitive performance without relying on arbitrary cutoffs while accounting for age and education, ensuring a more individualized assessment. Additionally, they mitigate ceiling and floor effects, particularly for high MMSE scores, and provide an intuitive visual representation, facilitating clinical interpretation. Moreover, they tend to provide uniformity in test score interpretation among patients with different background characteristics.

Similar to pediatric growth charts, they allow individuals to be positioned on a standardized scale according to their age and education level. Any measurement falling within the gray zone or showing a decline between successive tests is considered potentially abnormal, warranting further evaluation. The CC-MMSE and CC-MoCA outperform traditional fixed cutoffs, demonstrating a superior ability to detect cognitive decline [3,5].

### 1.3. Limitation of Cognitive Charts to an Age Threshold

In the mathematical models developed, it has been established that test results as a function of age follow a second-degree relationship. Due to this parabolic shape, a threshold has been defined. The average performance reaches a peak near 60 years for the MoCA and 65 years for the MMSE, corresponding to the age at which scores are at their highest.

Before these ages, the parabolic model would predict an improvement in scores with aging, which does not align with clinical reality, where cognitive performance generally remains stable or experiences a slight decline before the onset of cognitive deterioration. This discrepancy previously prevented the use of Cognitive Charts for individuals below the threshold age.

In this study, we proposed a threshold corresponding to the peak age of the parabola. Below this limit, we assumed that test scores remained constant, but we aimed to verify this hypothesis by analyzing the actual variations in cognitive performance below this threshold. If these variations proved minimal, it becomes possible to extend the use of CCs to all ages by adjusting the age of younger individuals to the corresponding threshold.

## 2. Materials and Methods

### 2.1. Study Sample

We obtained two large MoCA and MMSE datasets from the NACC-UDS, a well-established database. These were selected based on their large sample sizes and similarities, including intact participants, standardized evaluations, consensus diagnosis, and longitudinal data with annual follow-ups. Participants were tested yearly for 6 years on both measures (see Table 1).

### 2.2. Statistical Analyses

Mathematical equations used to derive the CCs are described in detail in our previous papers [3,5]. Here, we adjusted the age of each of the younger individuals to the threshold (threshold means ‘the lowest value permitted by the CC model so the model remains valid’) value of 60 (CC-MoCA) or 65 (CC-MMSE). This adjustment does not alter the CC but rather restores each individual’s cognitive performance to the threshold age. For longitudinal tracking, this implied that a patient may have two scores at the same Standardized Age, if both ages of the patient were below the threshold. The rules regarding intervals applied similarly to the current method (i.e., the vertical descent should not exceed the intervals already established on the CC to remain in a normal state). We conducted a performance validation to ensure suitability of this approach using CCs for younger elderly individuals. We compared the average MoCA and MMSE scores between individuals below the age threshold and those at the age threshold. Additionally, we performed a thorough analysis to assess the influence of age on the QuoCo scores and measured its impact below the age requirement. We employed linear regression analysis to examine the relationship between QuoCo and age for both the MoCA and MMSE assessments, specifically focusing on younger individuals. We compared this regression analysis with that of individuals above the age threshold. We evaluated the statistical performance (SE, SP, and Youden’s index) of the tool and, specifically, patients older than the threshold for tool use. Youden’s index, which combines both the SE and SP of the test, served as a fair comparison point for our analysis. We postulated that, if performances were comparable or superior, the method was valid and CCs could be used for younger patients.

## 3. Results

There were only minimal differences between individuals under 60 years old and those aged 60 years on the CC-MoCA. The mean MoCA score for individuals under 60 was 27.2 ± 2.4 (n = 587), while those aged 60 scored slightly higher with a mean score of 27.4 ± 2.4 (n = 94). Additionally, the variation in QuoCo-MoCA scores was found to be very low in individuals below the age limit.

**Figure 3:**
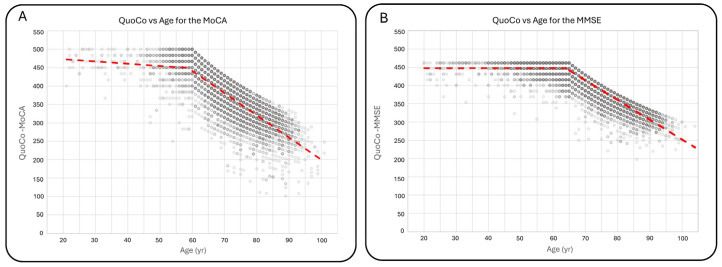
Variations in QuoCo-MoCA scores (**A**) and QuoCo-MMSE scores (**B**) as a function of age. This figure illustrates the determination of scores’ variations for ages below and above the threshold in QuoCo analysis. For individuals younger than the threshold, the age is set at the minimum age limit for QuoCo calculation. The graph demonstrates that the variation in QuoCo scores based on age is minimal in the case of QuoCo-MoCA (**A**) and exhibits a very low variation in the case of QuoCo-MMSE (**B**). These findings align with expectations, suggesting that both MMSE and MoCA scores show minimal age-related changes for individuals below the minimal age limit. Notably, these slopes are much smaller compared with those observed in individuals older than the threshold age. The dotted lines represent the separate linear regression curves established for the younger and older individuals up to the threshold age. The term threshold means ‘the lowest value permitted by the CC model so the model remains valid’.

For individuals under 60 years old, there was a decrease of 0.609 ± 0.200 QuoCo per year compared with a decrease of 6.025 ± 0.066 QuoCo per year for individuals over 60 years old. For the MMSE, there were no significant differences in scores between individuals under 65 years old and those aged 65 years. The mean MMSE score for individuals under 65 was 29.1 ± 1.2 (n = 2936), comparable to the score of 29.1 ± 1.3 (n = 482) for individuals aged 65 years. QuoCo score analysis based on age further supported these findings, showing minimal variation in QuoCo scores for the MMSE in individuals below the age limit. The rate of QuoCo-MMSE variation with respect to age under 65 years old was +0.00267 ± 0.00758 QuoCo per year, while for the group aged 65 years and older, it was −5.396 ± 0.029 QuoCo per year. Figure 3 illustrates the QuoCo scores for different age groups, highlighting the age-related trends in cognitive performance for the MoCA (Figure 3A) and for the MMSE (Figure 3B).

The statistical performance of the CC-MoCA was excellent. For the initial evaluation, CCs resulted in a slightly higher Youden’s index (0.67 vs. 0.65) for individuals under 60 years old compared with those aged 60 years and older. The SE was higher (0.95 vs. 0.84), while the SP was lower (0.73 vs. 0.81) in the younger group. Similarly, when analyzing transitions, CCs exhibited a higher Youden’s index (0.77 vs. 0.68) for individuals under 60 years old. In this case, both the SE (0.81 vs. 0.79) and the SP (0.96 vs. 0.89) were higher for individuals under 60 compared with those aged 60 and older. Similar findings were noted for the CC-MMSE.

At the initial evaluation, the Youden’s index was comparable between age groups, slightly lower for individuals under 65 (0.72 vs. 0.73). The SE was lower (0.74 vs. 0.76), while the SP was equal (0.98 vs. 0.98). In terms of transitions, the CC-MMSE demonstrated a significantly higher Youden’s index (0.74 vs. 0.58) for individuals under 65 years old. Additionally, both the SE (0.80 vs. 0.67) and the SP (0.94 vs. 0.91) were higher in this group compared with those aged 65 and over.

**Figure 4:**
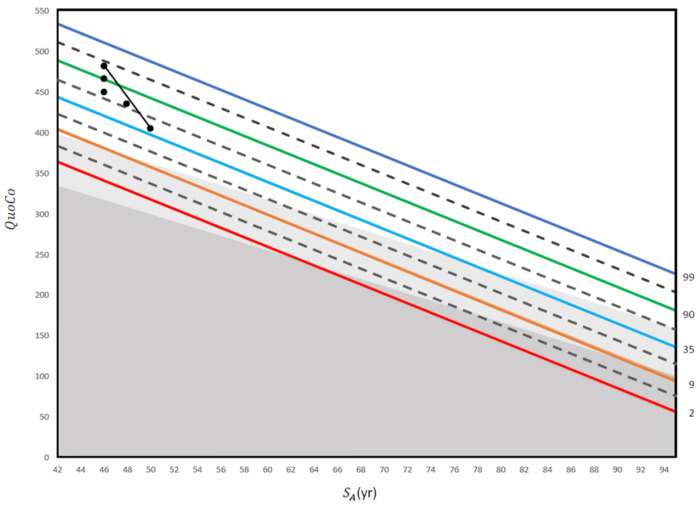
This figure illustrates a practical application of repeated screening tests using the MoCA in an electrician with 14 years of education. The first three QuoCo points of data were obtained at 50 years old (29/30), 55 years old (28/30), and 60 years old (27/30). Although the patient’s score only dropped from 29/30 to 27/30 between 55 years old and 60 years old, his trajectory has crossed one half interval and he is therefore within the MCI category (his performance has transitioned from normal to MCI; see [5]). Then, another QuoCo data point was taken at 62 years old where he performed at 27/30 on the MoCA (performance remains in the MCI category). Finally, the last QuoCo data point is 26/30 at 64 years old and here, the patient has transitioned from MCI to dementia (confirmed by further investigations) (see [5] for interpretation of the CC). This case illustrates that had we not used the data points before 60 years old, we would have missed the cognitive decline in this patient. In addition, many clinicians would be satisfied with 26/30 at 64 years old because they use a cutoff instead of a value that considers age and education, a dangerous shortcut in cognitive testing (see [5]). QuoCo scores are calculated automatically at quoco.org using age, education, and the MoCA score out of 30. QuoCo scores and Standardized Age (SA (yr)) were calculated automatically at quoco.org using age, education, and the MoCA score out of 30. Percentile lines are colored for easy read only.

A clinical case vignette is presented in Figure 4.

## 4. Discussion

A global health challenge remains the early screening, accurate identification, and proper management of MCI or dementia [10,11,12]. Although some primary care providers are at ease diagnosing and managing patients with cognitive changes, many physicians refer patients to neurologists, geriatricians, or geriatric psychiatrists. Evidence demonstrates that early diagnosis is associated with meaningful medical advantages [13,14] as well as reduced health care costs [15,16]. Barriers to early screening are multifactorial, but a major one is the lack of training and confidence physicians have in interpreting cognitive performances. In turn, significant cognitive changes as individuals age are not identified quickly, delaying diagnosis and treatments. Use of both CCs we developed for the MMSE and MoCA can facilitate this process (see quoco.org) [3,5].

The aim of this study was to validate the applicability of CCs in a younger population to expand the use of the CC-MoCA and CC-MMSE to the entire age span without any increased statistical error. To do so, we adjusted the age to the threshold when the actual data indicated that the patient was younger than the threshold. To validate this approach, we conducted a comprehensive analysis of the MoCA and MMSE assessments, along with their corresponding QuoCo scores. We also evaluated the statistical performance of CCs by comparing younger and older individuals. Our findings indicate minimal differences in MoCA scores between individuals under 60 years old and those aged 60 years, suggesting that cognitive performance remains relatively stable within these age groups. Similarly, the MMSE scores showed a comparable pattern between individuals under 65 years old and those aged 65 years, further supporting the stability of cognitive performance for younger individuals.

Analysis of QuoCo scores revealed a weak variation with age for individuals below the age threshold. This finding suggests that scores obtained at a younger age within these groups can be reasonably extrapolated to the threshold age, supporting the reliability and suitability of using CCs to track cognitive changes in younger individuals. Statistical analyses for the CC-MoCA and CC-MMSE at initial evaluation demonstrated comparable performance in terms of Youden’s index between individuals below and above the age threshold. These findings suggest that CCs are equally effective in detecting cognitive changes in younger individuals compared to their older counterparts.

When examining transitions, CCs employed in the younger age group exhibited superior performance compared with individuals above the threshold, highlighting their suitability for detecting cognitive changes in younger individuals and, thus, serving as a valuable tool for tracking cognitive functioning over time. This higher specificity among younger patients was described before in populations with a lower prevalence of cognitive disorders [10].

Altogether, our results suggest that CCs (see quoco.org) are a reliable and effective tool for evaluating cognitive performance in individuals across the entire age span. Indeed, their performance was found to be comparable or even superior when applied to younger patients, both in terms of initial evaluation and transitions. By utilizing CCs, healthcare professionals can gain valuable insights into their patients’ cognitive status and improve their ability to detect changes in cognitive function over time (see Figure 4 for a clinical case example).

It is difficult to compare our work with previous findings in the literature because, to our knowledge, no scientific group has developed similar cognitive curves. Clinical neuropsychologists typically compare performance over time from one assessment to another, but never by using such a sophisticated statistical methodology as our CCs. We can observe that our findings are in concordance with previous studies on the MMSE and MoCA in patients younger than 60 years old [17,18,19,20,21]. We also found that CCs cannot overcome the ceiling effect inherent to the cognitive tests used to develop the curves [3,5]. The development of CCs is based on linear regression models applied to large longitudinal databases, allowing for characterization of normal cognitive score trajectories according to age and education. The two fundamental concepts that emerged from our modeling are the Cognitive Quotient and Standardized Age. Data analysis from thousands of observations over decades revealed that the Cognitive Quotient follows a normal decline that is linearly associated with Standardized Age, making it a valuable tool for longitudinal cognitive performance monitoring (see quoco.org).

Perhaps one group explored cognitive functions in a similar way to us [22]. Scharre and colleagues compared longitudinal Self-Administered Gerocognitive Examination (SAGE) test scores to non-self-administered MMSE scores in five different diagnostic subgroups using a cohort of 665 consecutive patients. SAGE detected MCI conversion to dementia at least 6 months sooner than the MMSE. The process appeared to remove some barriers associated with performing cognitive assessments.

### Advantages, Limitations, and Future Directions

One of the main advantages over traditional methods is that our CCs allow for precise longitudinal monitoring of cognition without using arbitrary cutoffs. Moreover, CCs mitigate ceiling and floor effects, particularly for high MMSE scores. They further provide a powerful visual representation that facilitates understanding by patients and their families. Finally, they provide uniformity in test score interpretation among patients with different background characteristics.

A significant limitation to this work is the lack of normative data and large databases on minorities. Future research should focus on accumulating data from diverse ethnic groups. Also, future validation is necessary before we can conclude on the generalizability of the charts. Finally, future endeavors using computerized home-based assessments can be envisioned for longitudinal tracking of cognitive changes [23].

## 5. Conclusions

CCs now offer a promising avenue for tracking cognitive changes along the entire age continuum (see quoco.org). Our tool has the potential to enhance clinical decision-making and improve early treatment of degenerative conditions before irreversible damage. The number of people living with dementia is increasing around the globe, and policy makers must ensure there are strategies for early identification of dementia. Addressing risk factor levels at an early stage and promoting simple and efficient strategies such as the CCs for the early identification and tracking of longitudinal decline are crucial. New practice guidelines for the diagnostic evaluation of cognitive impairment due to AD or related dementia further encourage strategies such as the CC-MMSE and CC-MoCA [23].

## 6. Patents

Christian Gourdeau, Patrick Bernier, and Robert Laforce hold a patent for the CC methodology.

## Figures and Tables

**Table 1:** Characteristics of the study population for the MoCA and MMSE from the NACC database.

Characteristics	Healthy Controls	Mild Cognitive Impairment	Incident Dementia	Prevalent Dementia
***MoCA***				
*Participants < 60 years old* (*n* = 1007)	*n* = 577	*n* = 129	*n* = 14	*n* = 287
Age at the start of the study, year	51.59 ± 8.33	53.39 ± 6.99	53.14 ± 7.47	54.34 ± 5.25
Education, year	16.06 ± 2.45	15.76 ± 2.89	15.07 ± 2.59	14.84 ± 2.85
MoCA score at the start of the study	27.24 ± 2.40	23.94 ± 3.73	20.64 ± 3.95	14.01 ± 7.01
MoCA score at the end of the study	27.34 ± 2.34	23.97 ± 3.80	17.00 ± 6.64	12.99 ± 7.24
*Participants* ≥ *60 years old* (*n* = 9684)	*n* = 5168	*n* = 2015	*n* = 194	*n* = 2308
Age at the start of the study, year	73.66 ± 77.67	75.59 ± 7.96	76.82 ± 7.70	75.32 ± 14.67
Education, year	16.24 ± 2.77	15.94 ± 2.97	16.35 ± 3.09	15.55 ± 3.12
MoCA score at the start of the study	26.11 ± 2.88	22.55 ± 3.56	20.95 ± 3.54	14.67 ± 6.00
MoCA score at the end of the study	26.13 ± 2.94	22.27 ± 3.68	18.06 ± 4.39	14.67 ± 6.00
**MMSE**				
*Participants < 65 years old* (*n* = 5095)	*n* = 2712		*n* = 22	*n* = 2361
Age at the start of the study, year	56.60 ± 7.85		57.05 ± 8.97	57.96 ± 5.49
Education, year	16.39 ± 6.73		14.77 ± 3.19	16.34 ± 11.72
MMSE score at the start of the study	29.18 ± 1.18		28.59 ± 1.37	19.96 ± 7.16
MMSE score at the end of the study	29.21 ± 1.19		20.45 ± 7.06	16.15 ± 8.46
*Participants* ≥ *65 years old* (*n* = 16,774)	*n* = 7076		*n* = 533	*n* = 9165
Age at the start of the study, year	74.74 ± 7.03		79.60 ± 6.88	77.41 ± 7.02
Education, year	15.90 ± 6.17		15.47 ± 7.06	14.66 ± 8.40
MMSE score at the start of the study	28.83 ± 1.47		28.27 ± 1.74	19.66 ± 6.87
MMSE score at the end of the study	28.73 ± 1.59		22.27 ± 5.59	16.88 ± 7.66

## Data Availability

All data used in this study are available on the NACC website: https://naccdata.org/data-collection/forms-documentation/uds-3 accessed on 15 January 2023.

## Supplementary Materials

The following supporting information can be downloaded at: https://www.mdpi.com/article/10.3390/brainsci15040327/s1, Supplementary S1: Derivation of the model; Supplementary S2: Relationship between age and the MMSE and education and the MMSE.

## Abbreviations

The following abbreviations are used in this manuscript:

CC	Cognitive Charts
AACD	Age-associated cognitive decline
MMSE	Mini-Mental State Examination
MoCA	Montreal Cognitive Assessment
MCI	Mild cognitive impairment
NACC-UDS	National Alzheimer’s Coordinating Center—Uniform Data Set
AD	Alzheimer’s disease
ROC	Receiver Operating Characteristic
SE	Sensitivity
SP	Specificity
CI	Confidence interval
QuoCo	Cognitive Quotient
SA	Standardized Age
