# Supplementary material for: Expanding the Applicability of Cognitive Charts to the Entire Age Span
Source: Brain Sci. 2025 Mar 21;15(4):327. doi: 10.3390/brainsci15040327 (PMC12026361; doi:10.3390/brainsci15040327)

Supplementary S1. Representative cases.

We present herein a variety of representative cases examples taken directly from the CSHA dataset illustrating practical use of CC.

1. Case A.

A 76-year-old female is brought to her physician by her son because of memory problems. She has a Grade 10 education. Previously, a baseline MMSE (29/30) was completed at age 67. The physician calculated her QuoCo = 29/67 x 1,000 = 433 (QuoCo = MMSE / age x 1,000), and her S_A = 67 – 0.5 x 10 = 62 (S_A = age – 0.5 x years of education). These results were used to plot her performance on the CC (see Figure below). Later at 74 years old she had another MMSE (28/30) and calculations of her QuoCo (378) and S_A (69) were also charted on her CC. The overall profile of decline is acceptable since she remains within one percentile interval zone. She presents today at 76 years of age and again, her MMSE is completed indicating a performance of 26/30 (QuoCo=342, S_A =71), that is only 2 points below her previous performance. As shown on her CC, however, this last performance represents a total decline greater than 1.5 percentile interval zone from baseline (we recommend that the clinician interprets the general longitudinal decline by tracing a line from baseline to most recent data), and therefore is probably abnormal. Her physician also queries about functional abilities. Her son indeed reports mild decline in her ability to manage her finances and to cook. Further investigation was recommended. The patient was eventually referred to a memory clinic where mild AD was diagnosed.

	Age	Education	MMSE	S_A	QuoCo
1	67	10	29	62	433
2	74	10	28	69	378
3	76	10	26	71	342

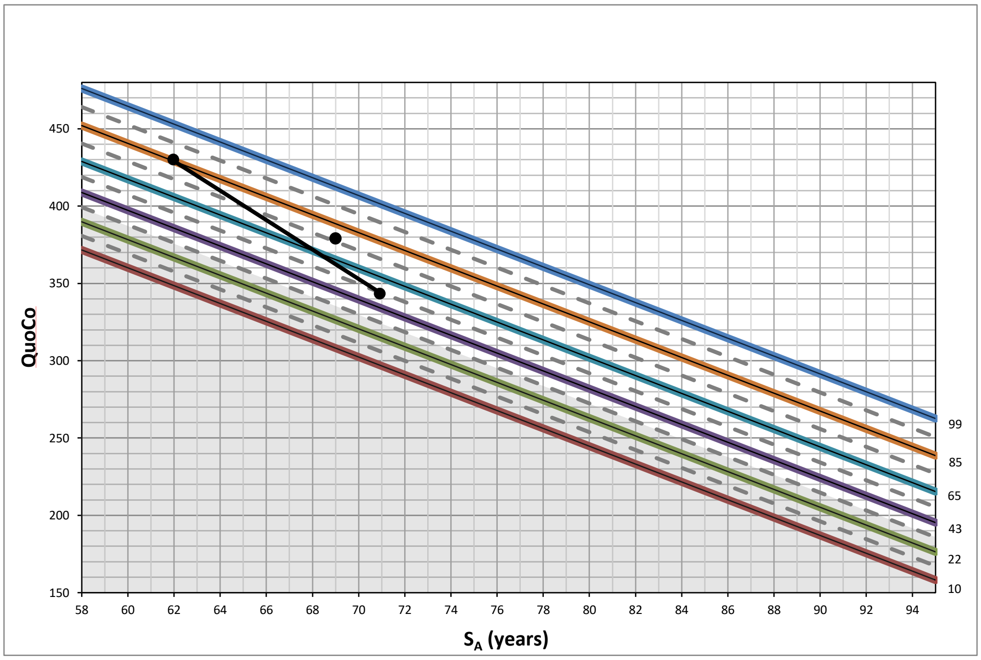

2. Case B.

This 89-year-old man was admitted to Geriatrics for repeated falls. During hospitalization, questions came up about his cognitive skills. His MMSE was 24/30. This result appeared low but his family explained that he had only completed 5 years of schooling. Looking into the patient's past medical charts, two previous MMSE performance were found both indicating 27/30 (one at 75 years old and the other at 81 years old). All of these were charted on the CC (75 years old: QuoCo=360, S_A=72.5; 81 years old: QuoCo=333, S_A=78.5; 89 years old: QuoCo=270, S_A=86.5) (see Figure below). Surprisingly, his profile remained within one percentile interval zone which was considered longitudinally normal. During recovery, his preserved cognitive skills emerged and he was scheduled for a six-month follow-up visit.

	Age	Education	MMSE	S_A	QuoCo
1	75	5	27	72.5	360
2	81	5	27	78.5	333
3	89	5	24	86.5	270

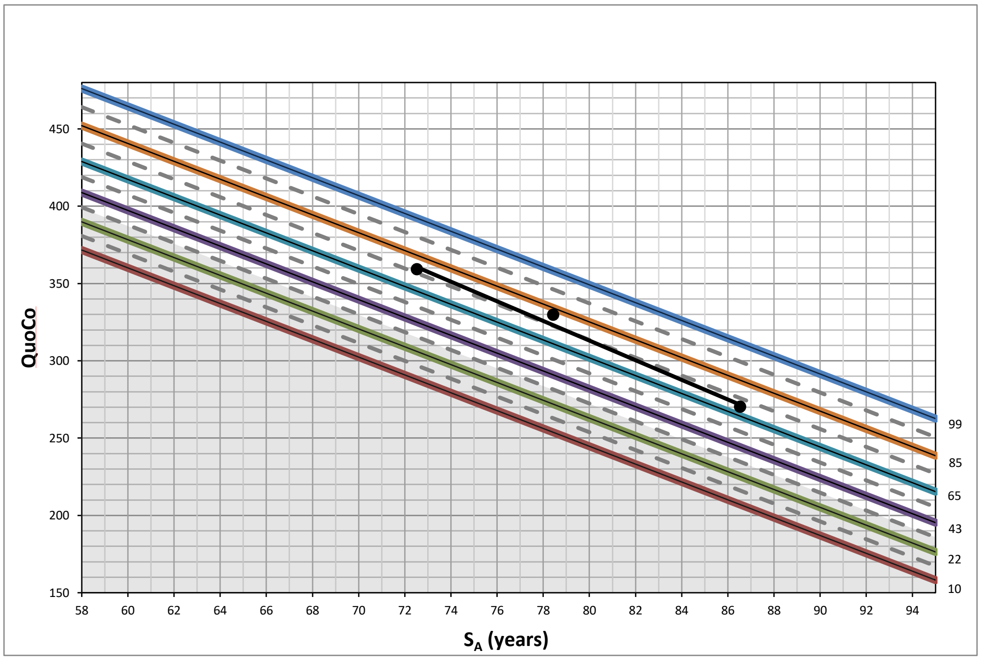

3. Case C.

Three patients were followed up on CC on three visits, at three year intervals, over a period of 6 years (see Figure below). The first case (left) is a 68 year-old female, the second (middle) is a 78 year-old male, and the last (right) is an 88 year-old male. Each completed 15 years of education and showed similar results on the MMSE on each of the three visits (28/30; 27/30; 26/30). Which patient is not following his expected cognitive decline? Answer: the patient on the left because she is crossing one percentile interval zone.

Patient 1					
	Age	Education	MMSE	S_A	QuoCo
1	68	15	28	60.5	412
2	71	15	27	63.5	380
3	74	15	26	66.5	351
Patient 2					
	Age	Education	MMSE	S_A	QuoCo
1	78	15	28	70.5	359
2	81	15	27	73.5	333
3	84	15	26	76.5	310
Patient 3					
	Age	Education	MMSE	S_A	QuoCo
1	88	15	28	80.5	318
2	91	15	27	83.5	297
3	94	15	26	86.5	277

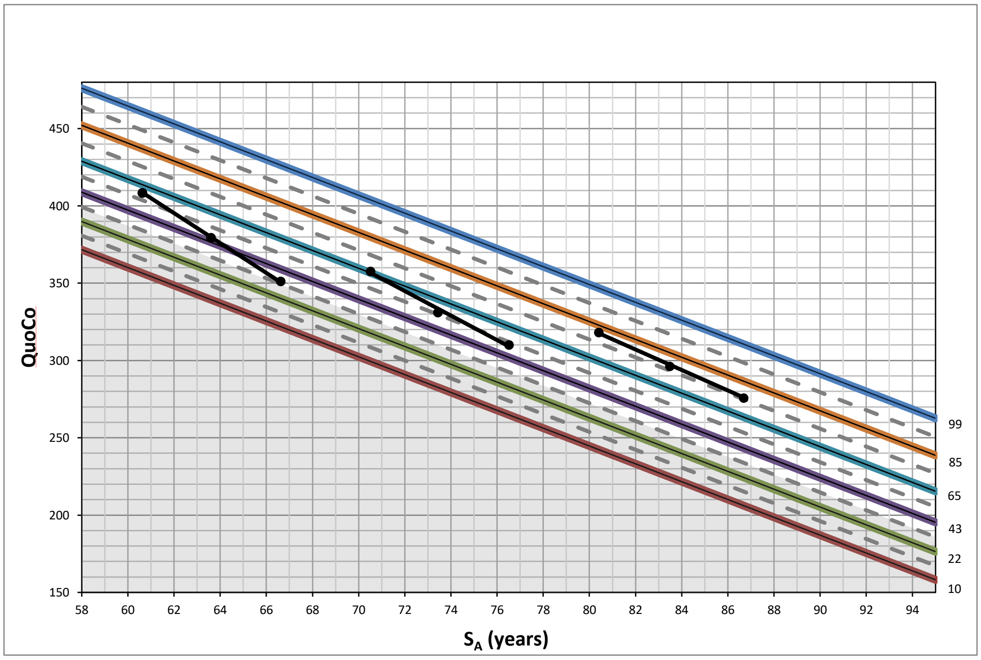

4. Six additional illustrative cases.

Cases A1, B1 and C1 all have normal trajectories. However, MMSE decline in the oldest patient (C1) reach as much as 4 points while MMSE in the younger (A1) patient remains stable. This illustrates that acceptable decline on MMSE varies depending on patient age.

Case A2 has 9 years of education. As age increases from 69 to 74 years old, with similar MMSE scores (29 and 28, respectively), trajectory is relatively unchanged even though education is much lower when compared to Case A1. When the MMSE drops from 28 at age 74 to 21 at age 79, CC reveals the anomaly. Of course, a drop of 7 points on the MMSE would have alerted any clinician but the CC further illustrate the severity of the phenomenon.

The two last cases (B2 and C2) show a situation where a cut-off of 24 on the MMSE is inappropriate. Indeed, both cases show an abnormal longitudinal trajectory despite staying above the proposed MMSE cut-off of 24/30. These cases provide additional examples of the strengths of our tool.

A_1

	Age	Education	MMSE
1	70	25	28
2	75	25	28
3	80	25	28

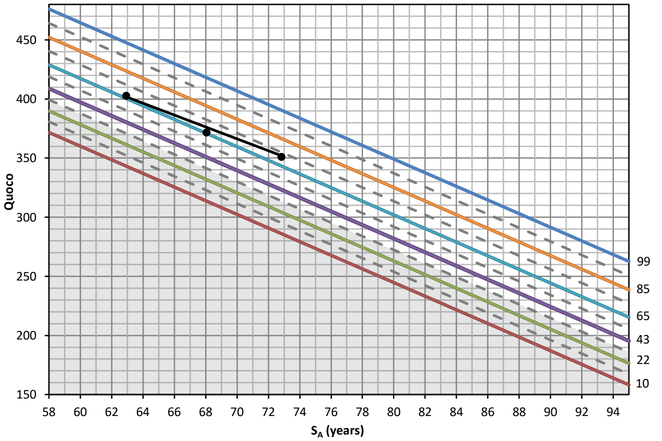

B_1

	Age	Education	MMSE
1	77	12	27
2	82	12	26
3	87	12	25

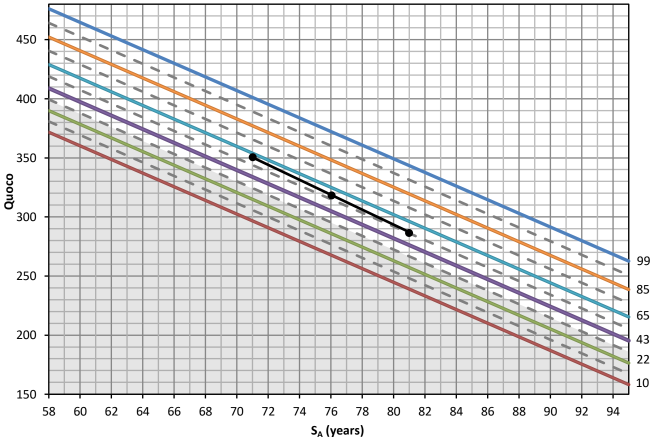

C_1

	Age	Education	MMSE
1	86	12	28
2	91	12	26
3	96	12	24

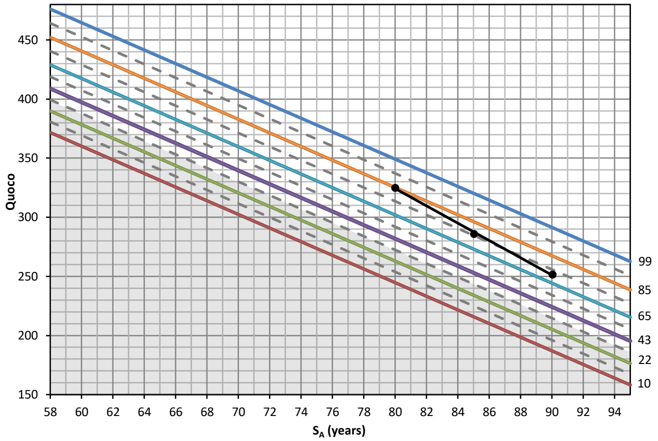

A_2

	Age	Education	MMSE
1	69	9	29
2	74	9	28
3	79	9	21

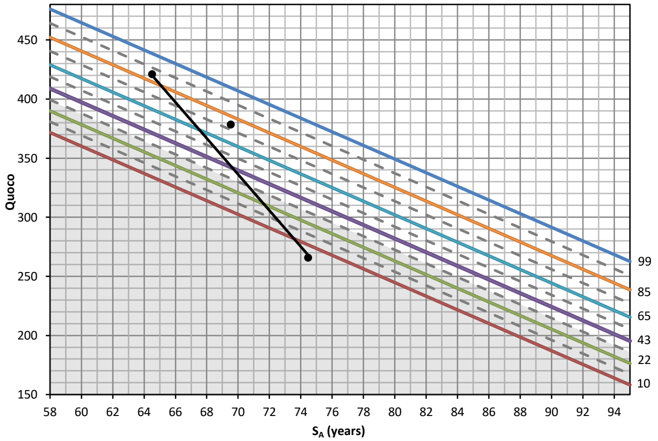

B_2

	Age	Education	MMSE
1	80	11	30
2	85	11	29
3	90	11	26

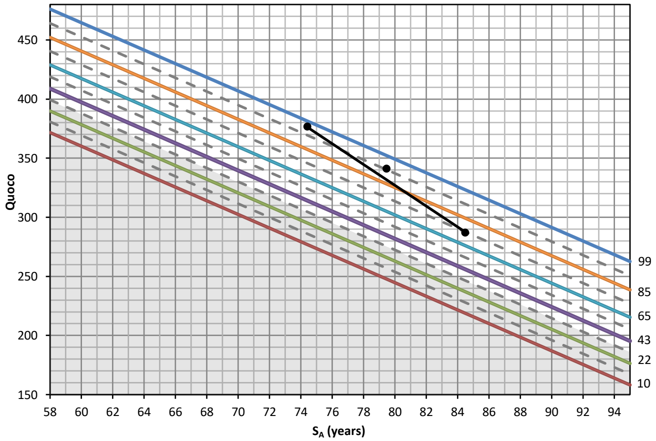

C_2

	Age	Education	MMSE
1	67	11	28
2	72	11	26
3	77	11	24

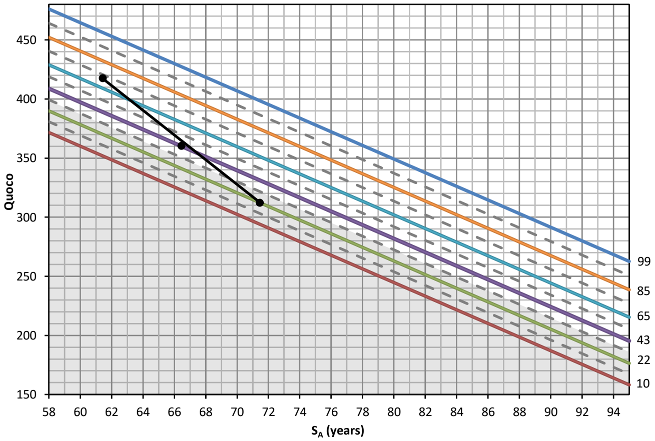

Supplement: Supplementary file 1 [file brainsci-15-00327-s001.zip › Supplementary S1.pdf]
