# Supplementary material for: Expanding the Applicability of Cognitive Charts to the Entire Age Span
Source: Brain Sci. 2025 Mar 21;15(4):327. doi: 10.3390/brainsci15040327 (PMC12026361; doi:10.3390/brainsci15040327)
Supplement: Supplementary file 1 [file brainsci-15-00327-s001.zip › Supplementary S2.pdf]

Supplementary S2. Clinical cases.

Case 1. What is worse, 26/30 or 21/30 on the MoCA?

Both members of this couple were referred to you for complaints of memory decline in the absence of functional decline. We have a 72 year-old mechanic with 11 years of education (Figure s1a) and a 69 year-old retired college professor with 18 years of education (Figure s1b). He got 23/30 while she got 30/30 on the MoCA. His score would raise concern if you apply the 26/30 cut-off but in the safe range on the Cognitive Charts (CC): S_A = age (max 100 years) - education (max 18 years) = 72 - 11 = 61, and QuoCo = MoCA score (max 30) / age (max 100 years) X 1000 = 23 / 72 X 1000 = 319 (Figure s1a) but hers cannot be better. Eight years later, the couple comes back with more cognitive concerns. The MoCA is readministered and she drops 4 points (26/30) while he drops 2 (21/30). It appears normal for her when using the recommended cut-off, but it is not when using the CC – MoCA (Figure s1b). Her cognitive trajectory declined more than one interval and this suggests possible early degenerative syndrome which was later confirmed on detailed history. She was diagnosed with mixed dementia (Alzheimer's and vascular) and therapy was promptly initiated as well as better control of risk factors. She was further placed on a list of eligible participants for research projects. By contrast, our gentleman remained within less than a half-interval from previous performance and did not show functional decline (Figure s1a). His score however, fell in the light-grayed zone which suggests that he may have Mild Cognitive Impairment (MCI). In his case, serial follow-ups separated by no longer than 12 months are recommended. In addition to reiterating the importance of serial reassessments, this case highlights the value of the CC-MoCA curves beyond using a simple cut-off.

Figure s1a

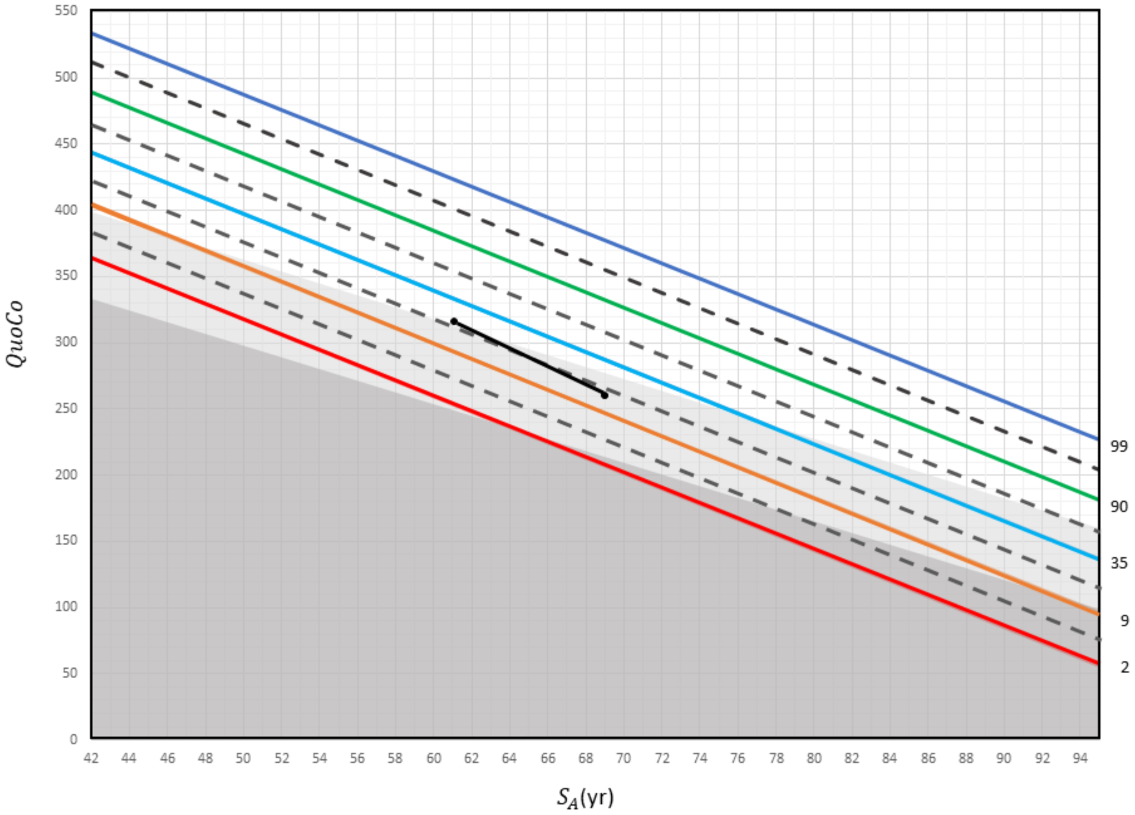
550
500
450
400
350
300
250
200
150
100
50
0
$QuoCo$
42
44
46
48
50
52
54
56
58
60
62
64
66
68
70
72
74
76
78
80
82
84
86
88
90
92
94
S_A(yr)
99
90
35
9
2

Figure s1b

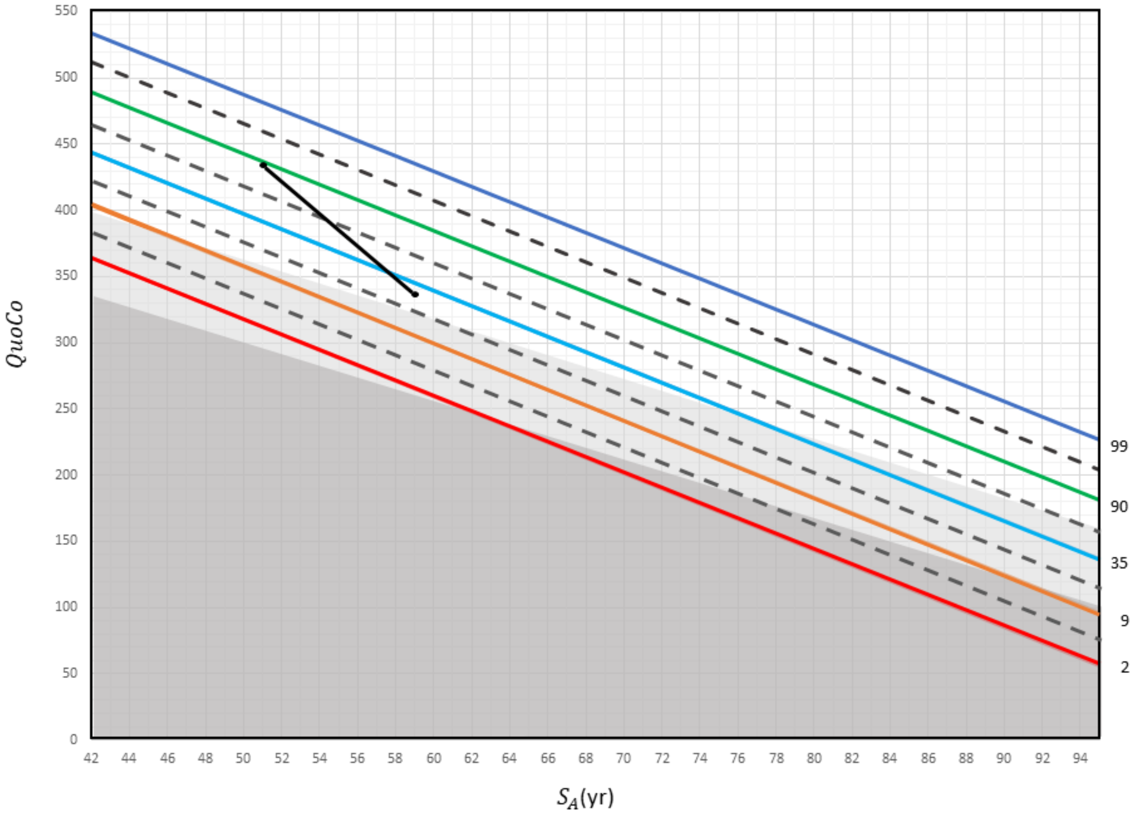

550
500
450
400
350
300
250
200
150
100
50
0
42
44
46
48
50
52
54
56
58
60
62
64
66
68
70
72
74
76
78
80
82
84
86
88
90
92
94
QuoCo
S_A(yr)
99
90
35
9
2

Case 2. Same scores but not the same outcome…

Let’s consider three patients:

Patient #1: 61 years old, 16 years of education, baseline MoCA = 29/30

Patient #2: 74 years old, 16 years of education, baseline MoCA = 29/30

Patient #3: 82 years old, 16 years of education, baseline MoCA = 29/30

They were followed up over a period of 6 years with a decline up to 26/30 on the MoCA.

Figure s2 demonstrates that even though they performed similarly on the MoCA, their trajectories are very different. Patient #1 (Figure s2a) shows a decline beyond one interval (two half-intervals = two changes in cognitive state that is from healthy control to MCI and from MCI to dementia), hence suggesting a neurodegenerative disorder. Patient #2 (Figure s2b) showed a decline beyond a half interval suggesting a change from healthy control to MCI. Patient #3 (Figure s2c) showed normal aged-associated cognitive decline.

Figure s2a

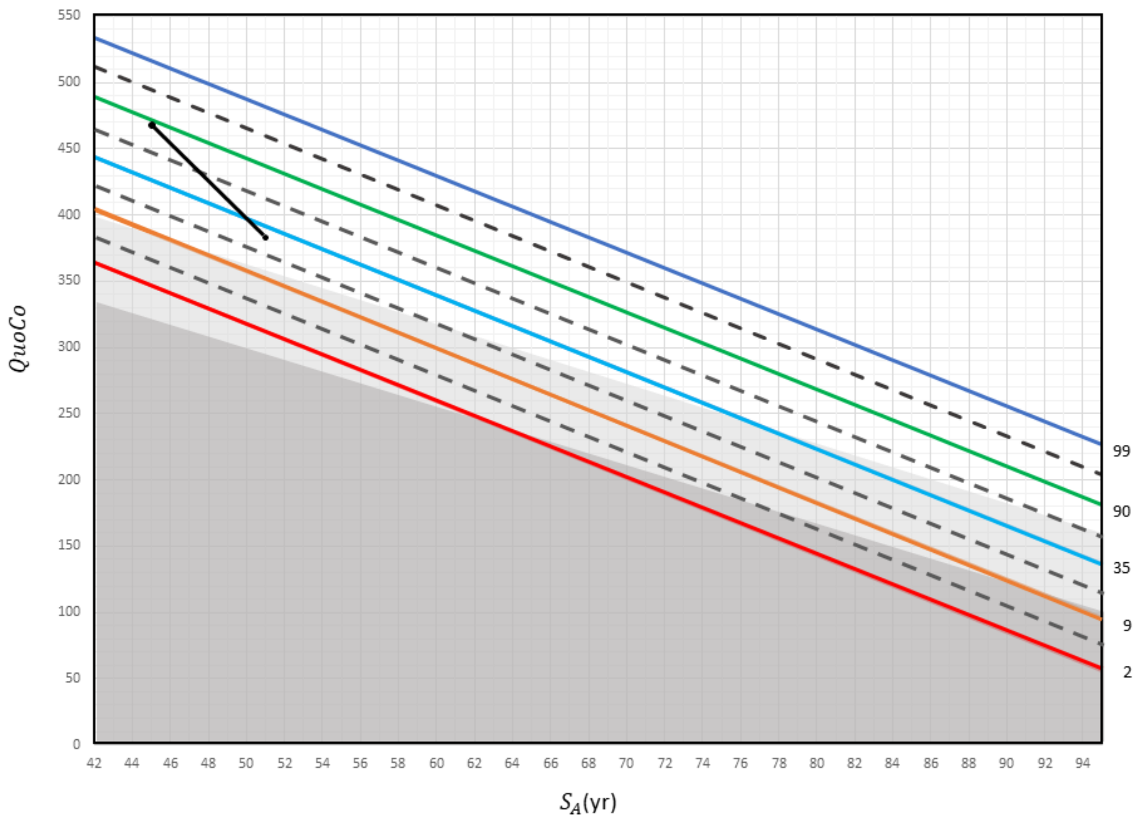
550
500
450
400
350
300
250
200
150
100
50
0
QuoCo
42 44 46 48 50 52 54 56 58 60 62 64 66 68 70 72 74 76 78 80 82 84 86 88 90 92 94
S_A(yr)
99
90
35
9
2

Figure s2b

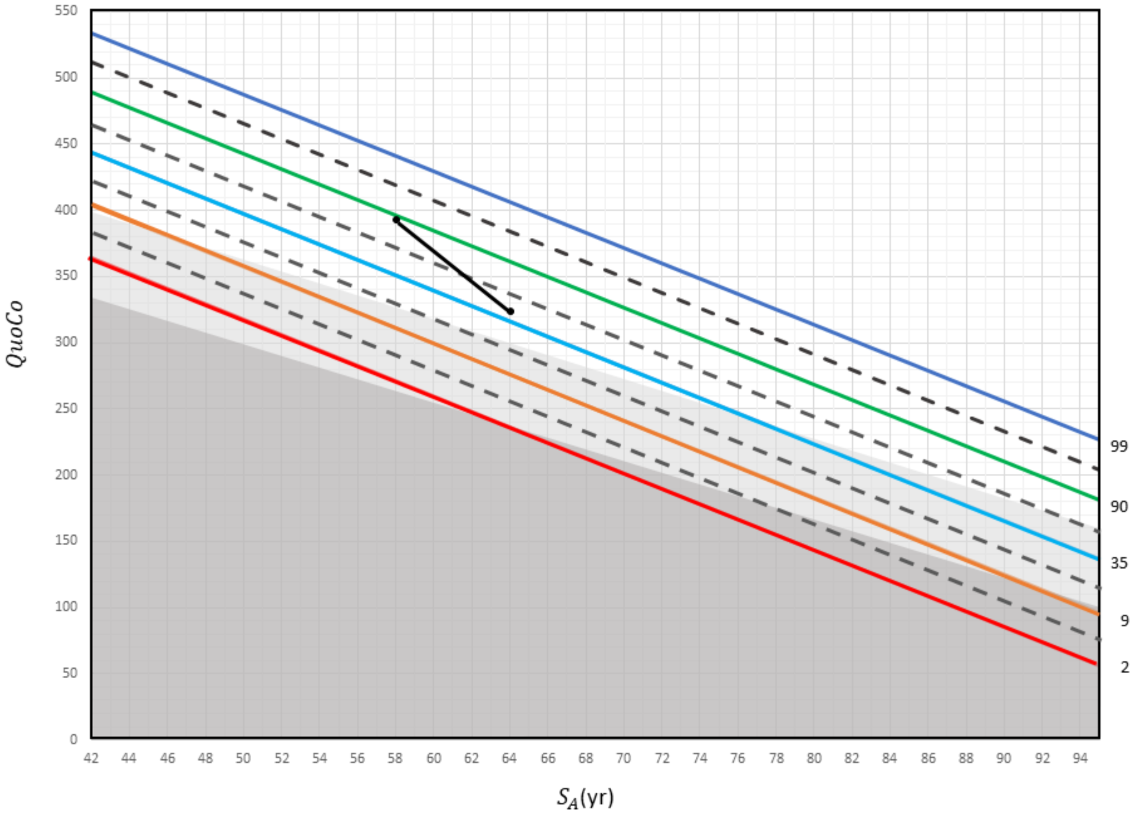

550
500
450
400
350
300
250
200
150
100
50
0
42
44
46
48
50
52
54
56
58
60
62
64
66
68
70
72
74
76
78
80
82
84
86
88
90
92
94
$QuoCo$
S_A(yr)
99
90
35
9
2

Figure s2c

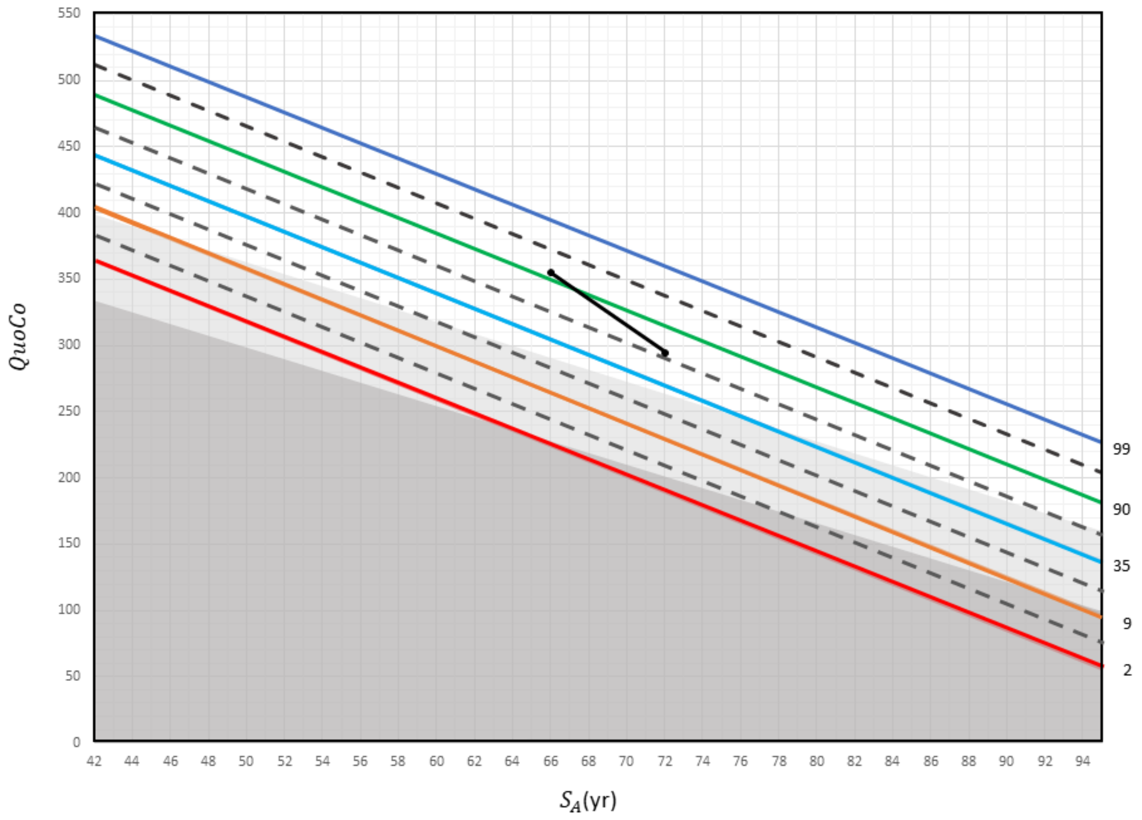

550
500
450
400
350
300
250
200
150
100
50
0
QuoCo
42
44
46
48
50
52
54
56
58
60
62
64
66
68
70
72
74
76
78
80
82
84
86
88
90
92
94
S_A(yr)
99
90
35
9
2
